# Inter-fraction movements of the prostate and pelvic lymph nodes during IGRT

**DOI:** 10.1007/s13566-018-0366-3

**Published:** 2018-11-28

**Authors:** Ulrika Björeland, Joakim Jonsson, Magnus Alm, Lars Beckman, Tufve Nyholm, Camilla Thellenberg-Karlsson

**Affiliations:** Department of Radiation Sciences, Umeå University, Sjukhusfysik, Sundsvallssjukhus, 85186 Sundsvall, Sweden

**Keywords:** Prostate, Lymph nodes, CTV, Movements

## Abstract

**Objectivities:**

The aim of this study was to evaluate inter-fraction movements of lymph node regions that are commonly included in the pelvic clinical target volume (CTV) for high-risk prostate cancer patients. We also aimed to evaluate if the movements affect the planning target volumes.

**Methods:**

Ten prostate cancer patients were included. The patients underwent six MRI scans, from treatment planning to near end of treatment. The CTV movements were analyzed with deformable registration technique with the CTV divided into sections. The validity of the deformable registration was assessed by comparing the results for individual lymph nodes that were possible to identify in all scans.

**Results:**

Using repetitive MRI, measurements showed that areas inside the CTV (lymph nodes) in some extreme cases were as mobile as the prostate and not fixed to the bones. The lymph node volumes closest to the prostate did not tend to follow the prostate motion. The more cranial lymph node volumes moved less, but still independently, and they were not necessarily fixed to the pelvic bones. In 95% of the cases, the lymph node motion in the R-L direction was 2–4 mm, in the A-P direction 2–7 mm, and in the C-C direction 2–5 mm depending on the CTV section.

**Conclusion:**

Lymph nodes and prostate were most mobile in the A-P direction, followed by the C-C and R-L directions. This movement should be taken into account when deciding the margins for the planning target volumes (PTV).

**Electronic supplementary material:**

The online version of this article (10.1007/s13566-018-0366-3) contains supplementary material, which is available to authorized users.

## Introduction

External radiotherapy of prostate cancer relies on the precise knowledge of the prostate location at treatment. Because the prostate is a mobile organ [1–3], and to minimize planning target volumes (PTV), radio opaque fiducial markers [4–9] are commonly used. Before treatment-planning computed tomography (CT), three or more fiducials are implanted into the prostate. Fiducials and bones are easily visualized at the time of treatment with cone beam CT (CBCT) or X-ray imaging (kV/MV-imaging) by using image-guided radiotherapy (IGRT).

For prostate cancer with lymph node involvement, the clinical target volume (CTV) includes both prostate and lymph nodes in the pelvic area [10–14], and acceptable nodal coverage when performing IGRT with prostate fiducials can be a challenge [15]. When defining the margins for lymph node irradiation, it is often assumed that the nodes are fixed to bones [16–19]. A study using magnetic resonance imaging (MRI) has shown that the lymph nodes follow the vessels rather than the bony anatomy [20]. Hinton et. al [21] pointed out that vessel mobility, and therefore lymph node mobility, relative to the prostate, needs to be considered. Evaluation of lymph node mobility directly has never been done to our knowledge.

Analyzing mobility with deformable image registration (DIR) is an effective tool because it offers the possibility to follow organ changes or contour propagation between imaging occasions [22]. It also allows dose tracking to account for organ deformation [23]. MRI is the ideal image input type due to excellent soft tissue contrast [24] for DIR analysis and a well-known open-source software such as elastix [25] allows many possibilities for image processing and registration.

In the present study, we investigated the mobility, using DIR, inside the pelvic clinical target volume (CTV). The CTV was divided into different sections, and the center of mass points (COM) were calculated for each section. Lymph nodes were identified in six MRI examinations distributed over the entire treatment period. The intra fractional movements were validated by the identification of the same lymph node at each imaging occasion, and these movements were compared to the deviation calculated with DIR.

## Methods

We used repetitive MRI for ten patients (*n* = 10) diagnosed with prostate cancer without evidence of lymph node involvement and scheduled for treatment with image-guided radiotherapy in this study. Normal patient preparation and treatment procedures at our clinic were applied, but with six additional MRI scans. Approximately 30 min before imaging, the patient was asked to empty his bladder and then drink 200–300 ml water. The baseline scan was acquired 4–7 days before start of the IGRT, and the following scans were taken on treatment days (TD) 1, 3, 5, 20, and 35 (TD1, TD3, etc.). The study was approved by the local ethical committee (dnr. 2013-3-31M).

### MRI scanning

The MRI scans were performed 10–15 min after treatment-planning CT (at baseline) or after IGRT. For patient nos. 1–5, the MRI scanner used was a Siemens Magnetom Espree 1.5T (T2 SPACE: TR/TE 2000/133 ms; 176 slices; FOV 300 mm, voxel size 1.2 × 1.2 × 1.0 mm^3^). For patient nos. 6–10, a GE Signa PET/MRI 3T (T2 CUBE SPACE: TR/TE 1240/103 ms; 196 slices; FOV 320 mm, voxel size 0.81 × 0.81 × 1.0 mm^3^) was used. All images were 3D-distortion corrected. All scans were performed with a flat table top and with the same fixation equipment as used during treatment.

### CTV and lymph node outlining in the MRI scans

Study-specific delineations were performed by the same radiation oncologist for all patients. The CTV was outlined according to RTOG guidelines [26, 27], and it included prostate, vesicles, and lymph nodes in the baseline scan. Specific lymph nodes were identified in the baseline MRI scan by an MRI radiologist. In total, 23 lymph nodes were identified with 1–4 lymph nodes per patient, and all were classified as normal with respect to size and shape. These lymph nodes were subsequently identified in the following MRI scans, enabling tracking of specific lymph nodes during the treatment period. All patients but one had six MRI scans and one patient had four scans. The center of mass (COM) of the lymph nodes was used as a validation structure for the deformable image registration.

### CTV sections for movement calculation

**Fig. 1:**
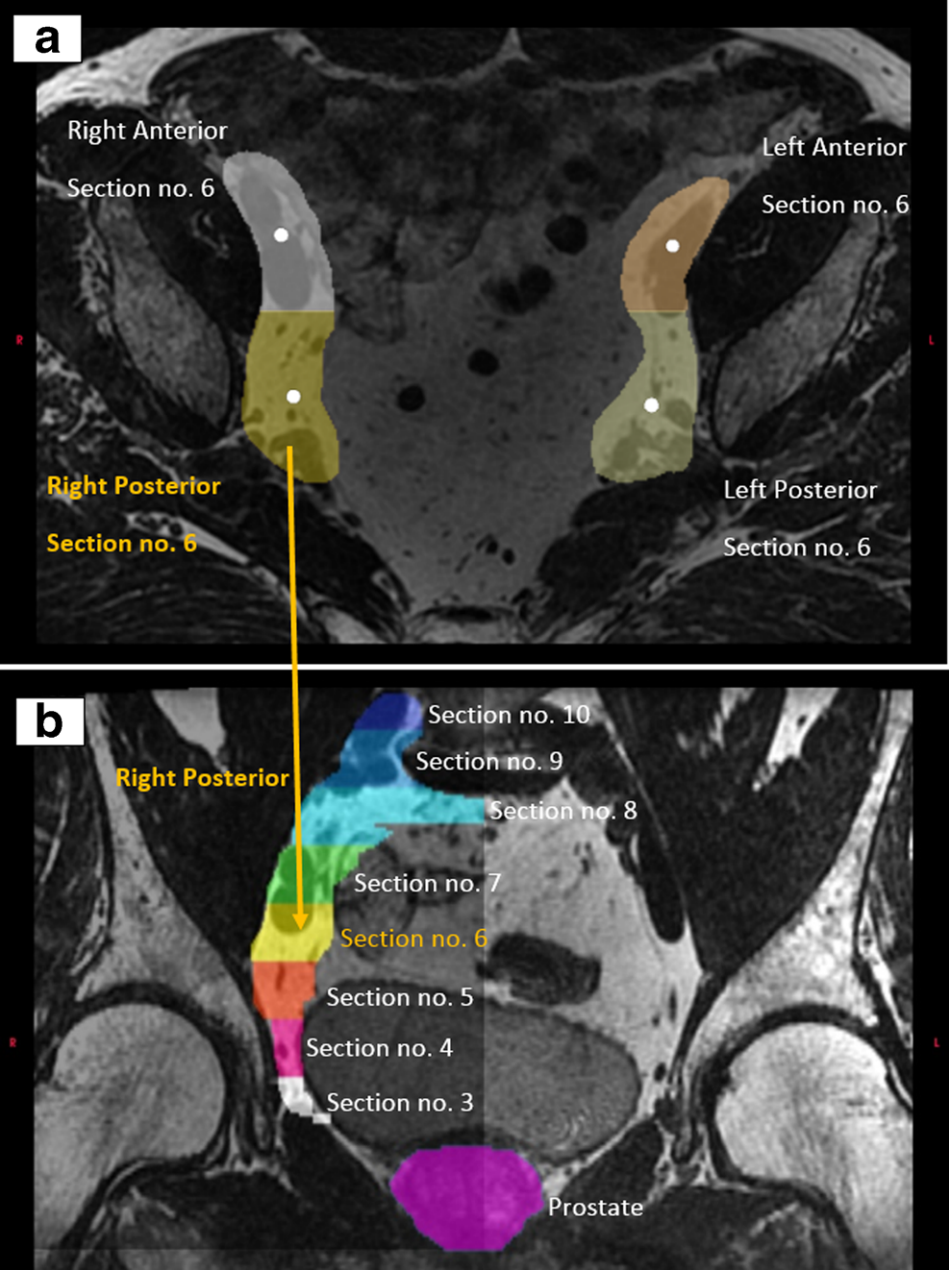
**a** Sections, with the COM point as a white dot, in the four CTV sub regions. **b** Sections no.10 to 3 in posterior right sub region, prostate occupies section nos. 1 and 2 for this patient. Note that the left posterior sections are not visualized in this image

The CTV covers a large volume within the patient. To evaluate movement within the CTV between imaging occasions, we sectioned the CTV as follows:

1. The CTV was divided in to four sub regions: anterior (right and left) and posterior (right and left), Fig. 1a in the following procedure:
  The center of mass was identified inside CTV in patient coordinate system. The COM point was the origin for the sub regions. To the right of COM, the right section; to the left of COM, the left section. Below the COM, the posterior section; and above COM, the anterior section.
2. The four sub regions were divided into ten sections along the Cranio-Caudal (C-C) direction, see Fig. 1b.

Each section included 1/10 of all transverse image slices covering the CTV. In total, up to 40 sections were defined (the number varied according to the different shapes of the individual CTV from patient to patient). In every section, the center of mass point was calculated to be used for the movement calculation in that specific section. The white dots in Fig. 1a represent the COM in different sub regions in section no. 6. In Fig. 1b, the right sub region is represented with the different sections and prostate.

The prostate was included in the CTV. The prostate was considered as one volume and was not divided into sub regions or sections.

### Image registration

**Fig. 2:**
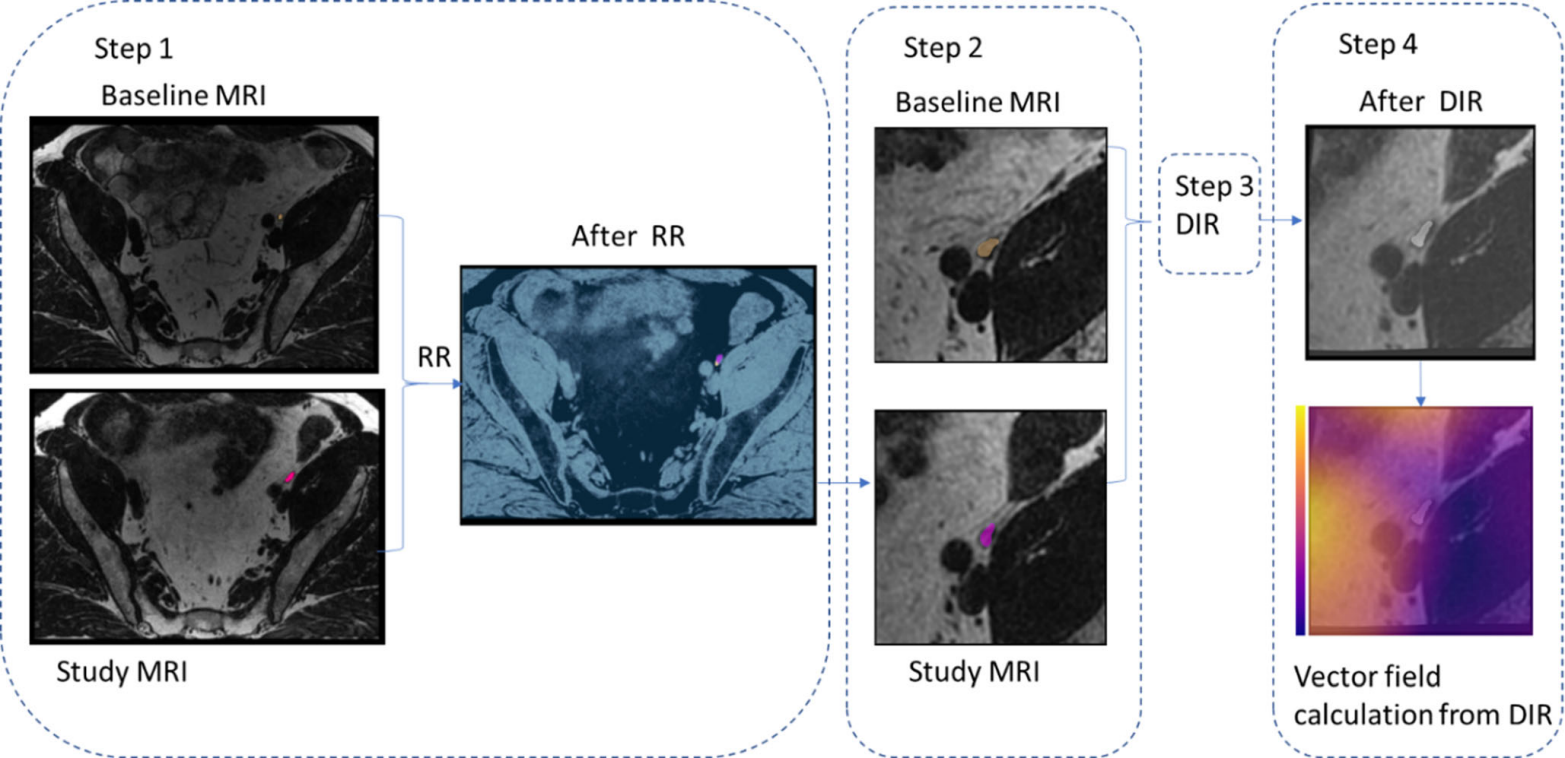
Image registration scheme. In step 1, lymph node in color in baseline respective study MRI. Rigid registration (RR) image is a normalized subtraction image, dark indicates perfect rigid registration. In step 2, a smaller cubical volume was extracted and upsampled around a point of interest in the baseline image or study (a lymph node or section center of mass). In step 3, the upsampled volumes was deformably registered (DIR). In step 4, the vector field in blue indicates 0 mm deviation from baseline and yellow 4 mm deviation from baseline. Deformed lymph node in white

The software package elastix [25] implemented in the MICE Toolkit (NONPI Medical AB, Umeå, Sweden) was used for all image registrations in the study. The elastix parameter files used can be found in the supplementary materials. The registrations were performed as follows (see also Fig. 2).

**Fig. 3:**
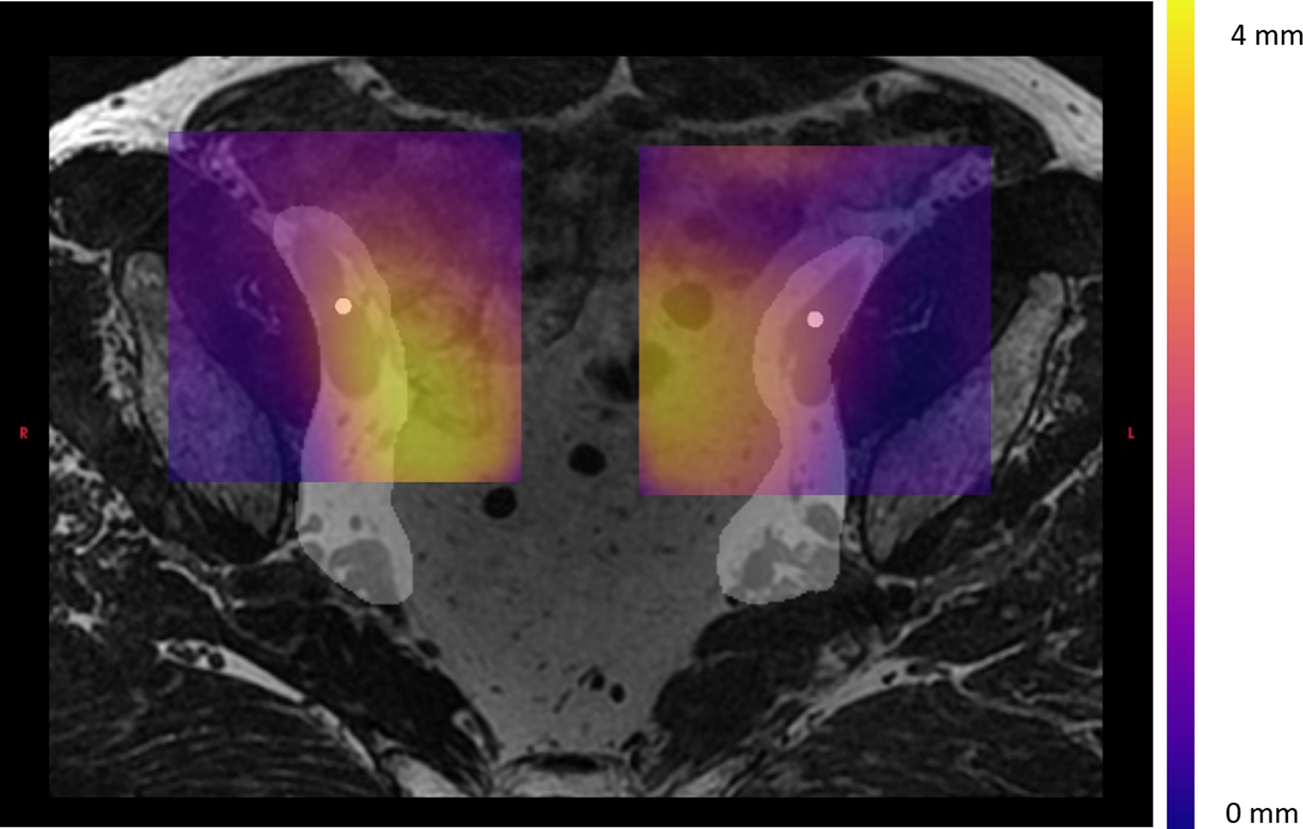
Vector fields showing deviation from baseline MRI in right anterior and left anterior in colors. The white structure is CTV and the white dot is COM

1. The study MRI was rigidly registered to the baseline MRI using a mutual information metric, taking the entire anatomy into account. This implies that the bony anatomy had a large influence on the registration result, and this registration will henceforth be referred to as the *bone* registration.

2. A smaller cubical volume (each side 70–90 mm) was extracted around a point of interest in the baseline image (a lymph node or section center of mass). This volume was upsampled to an isotropic resolution of 0.25 mm using a b-spline interpolator (order 3). The corresponding volume was extracted and upsampled in the bone registered study MRI
3. The upsampled volumes were then deformably registered using a mutual information metric and a b-spline (order 3) transform. For the calculations of the CTV movements, 12–16 subvolume registrations were performed per patient, depending on the patient size, and each subvolume covered 1–3 sections. The minimum volume evaluated with DIR parameters was 70 × 70 × 70 mm^3^ and maximum 90 × 90 × 90 mm^3^. COM were located inside the evaluated DIR volume and at least 15 mm from the DIR volume edges to avoid edge effects. If the COM point was closer than 35 mm to an end slice, the DIR calculation center was moved in the Cranio or Caudal (C-C) direction. For the calculations of lymph node movements, one registration was performed for each lymph node.
4. Movements were visualized in a 3D-vector field generated from the DIR (Fig. 3). The vector field represents the movement in each voxel from the baseline MRI to an MRI scanned at either TD 1, TD 3, TD 5, TD 20, or TD 35. The vector field is three dimensional and represents movements corresponding to Right-Left (R-L), Anterior-Posterior (A-P), or Cranio-Caudal (C-C) in the patient reference system.

### Validation procedure

To validate the accuracy of the deformable image registrations, 23 lymph nodes identified in all scans (TD1-TD35) were used. By comparing the position of a lymph node at baseline with a TD study after rigid registration, the movement caused by tissue deformation is known. This known deformation was compared with the result given by the DIR around that specific lymph node.

## Results

### Lymph nodes vs. COM points in the same section of the CTV

Twenty-three different lymph node movements were compared to the COM movement in the same section for all treatment day scans. This intra sectional movement indicated that the lymph node motion and the COM motion in the same section were similar, with a mean deviation between movements in the two points of 0.6 ± 0.7 mm. All lymph nodes were identified in section nos. 4–10. This result indicates that the COM point within a CTV section is a good surrogate marker for lymph node motion.

### Displacements from baseline

We use “COM” to include both COM points and lymph nodes throughout the result section.

**Fig. 4:**
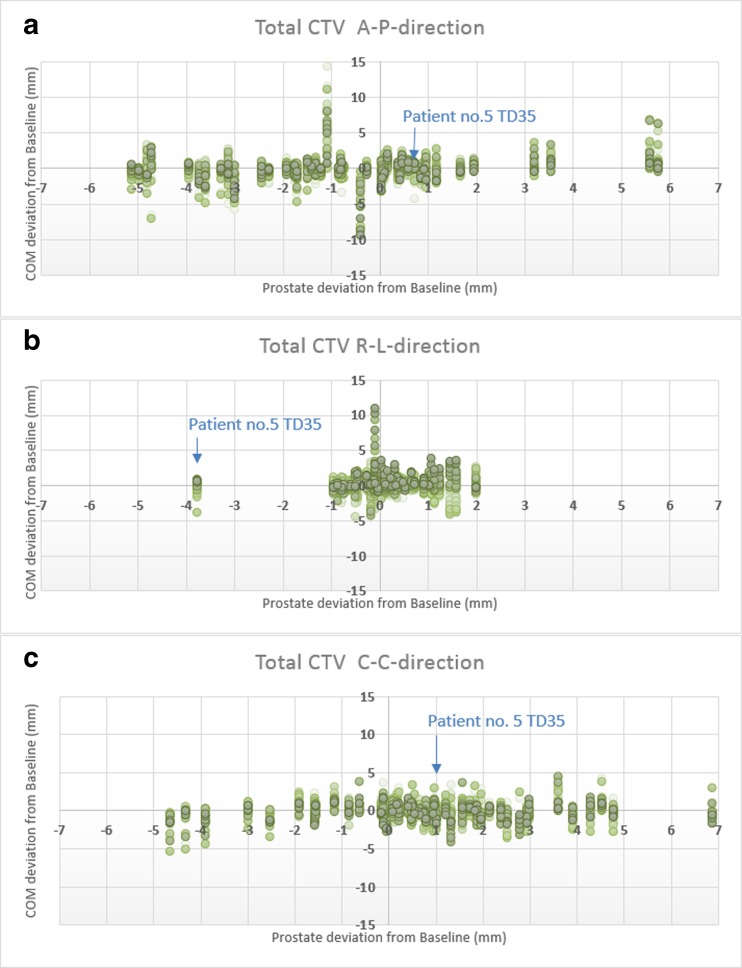
Prostate displacements from baseline in **a** A-P direction, **b** R-L direction, and **c** C-C direction for all patients. **a**–**c** One vertical data set represents one TD MRI COM points for one patient. The data points for patient no. 5 on treatment day 35 are indicated in blue text

**Fig. 5:**
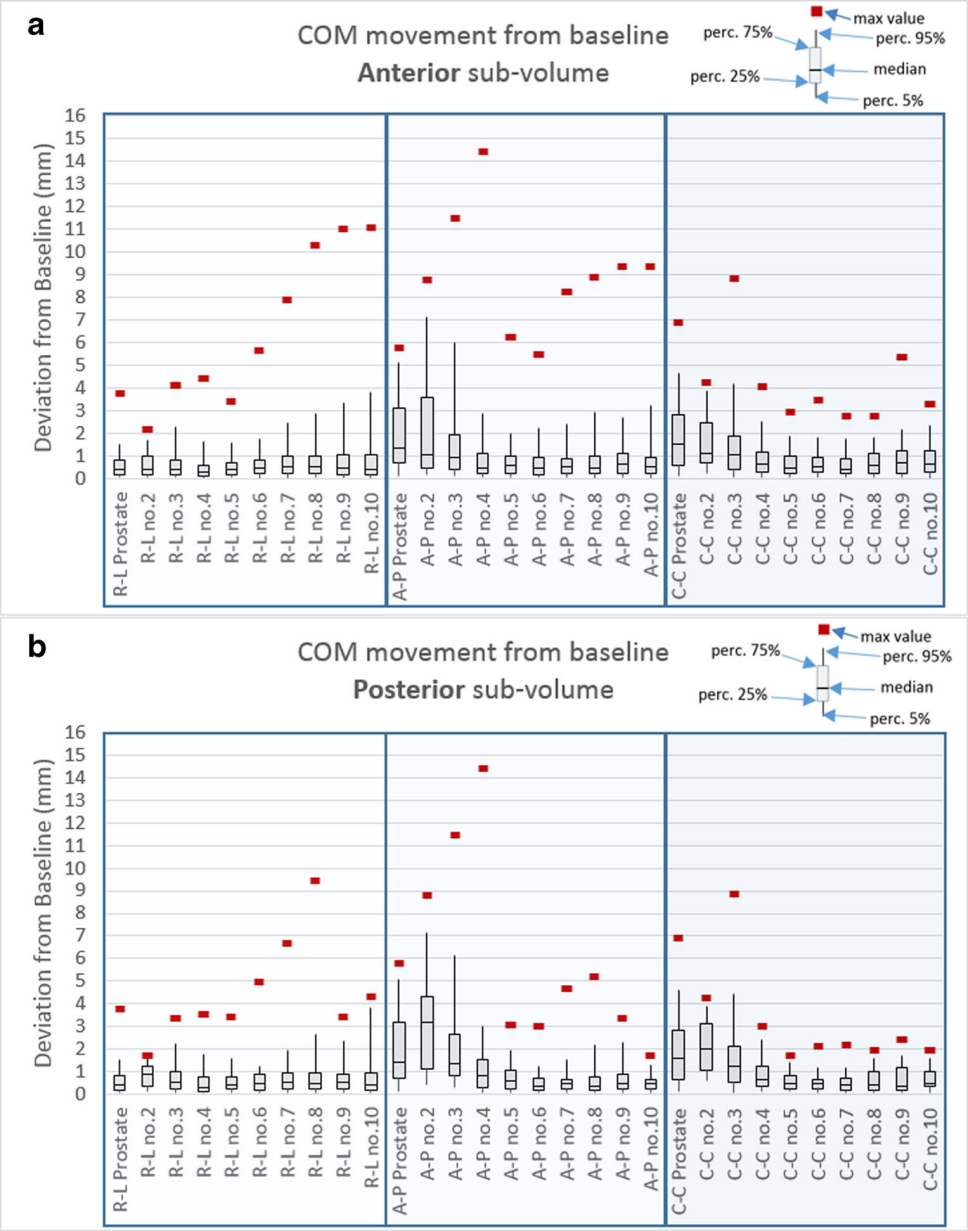
**a** Anterior sub-volume, deviations from baseline in different directions (R-L, A-P, C-C) for prostate and the sections in CTV. **b** Posterior sub-volume, deviations from baseline in different directions (R-L, A-P, C-C) for prostate and the sections in CTV

The displacements from baseline for the COM and prostate were investigated with DIR. In Fig. 4a–c, the displacement patterns in the A-P, R-L, and C-C directions are shown. Each prostate displacement corresponds to one patient’s TD displacement as compared with the COM. As an example, patient no. 5’s displacements at treatment day 35 are highlighted with a blue arrow. The extreme COM values are not shown in this figure but are presented in Fig. 5a, b.

**Fig. 6:**
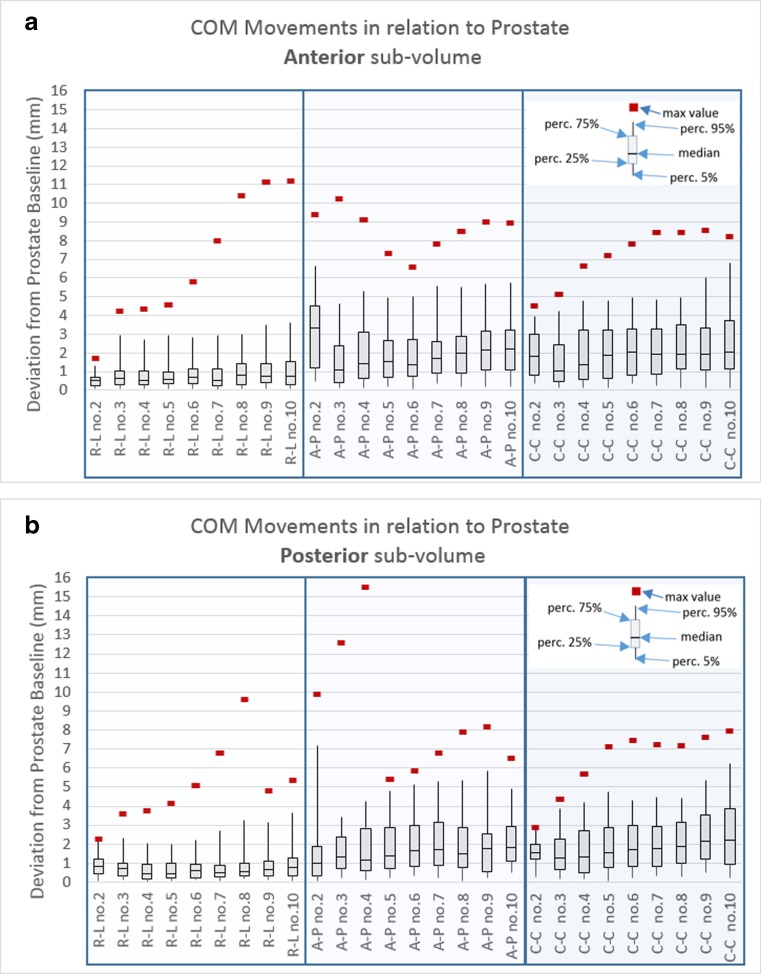
**a** Anterior sections and their relation to prostate movement. **b** Posterior sections and their relation to prostate movement

In Figs. 5 and 6, the median value of the deviation from the baseline is presented, as well as the 5%, 25%, 75%, and 95% percentile and the max deviation. Figure 5 shows the deviations from baseline in different movement directions for the prostate and COM of the sections in the CTV. The posterior sections were more mobile close to the prostate, and most pronounced in the A-P direction. The anterior sub-volume was less mobile but had larger max deviations. The COM mobility, which covered 95% of the movements, in the R-L direction were 1–4 mm; A-P, 1–7 mm; and C-C, 1–5 mm depending on the section and for prostate: R-L, 2 mm; A-P, 5 mm; and C-C, 5 mm (see Table 1). The absolute mean deviation varied in the different directions and sub-volumes, and were in the range R-L, 0.4–1.1 mm; A-P, 0.5–3.3 mm; and C-C, 0.5–2.1 mm for the different sections. For prostate, the absolute mean deviation were in R-L, 0.6 mm, A-P, 2.0 mm; and C-C, 1.9 mm. The standard deviation was close to or lower than 1 mm for most sections. For prostate and sections close to prostate, the standard deviation was closer to 2 mm in A-P direction, Table 1.

**Table 1:** Deviations from baseline in all the sections in 95% of the cases in mm and (absolute mean value ± sd) in mm

	Sec.	Prostate	No. 2	No. 3	No. 4	No. 5	No. 6	No. 7	No. 8	No. 9	No 10
Movement direction in the anterior or the posterior sub-volume (mm)	R-L ant	1.52 (0.59 ± 0.65)	1.23 (0.44 ± 0.48)	2.05 (0.59 ± 0.73)	1.08 (0.45 ± 0.66)	1.47 (0.55 ± 0.52)	1.87 (0.67 ± 0.72)	2.58 (0.87 ± 1.04)	3.04 (0.97 ± 1.27)	3.56 (0.99 ± 1.44)	3.64 (1.07 ± 1.73)
	R-L post		1.69 (0.83 ± 0.56)	2.25 (0.76 ± 0.72)	1.74 (0.54 ± 0.65)	1.59 (0.56 ± 0.52)	1.23 (0.59 ± 0.65)	1.93 (0.71 ± 0.82)	2.62 (0.82 ± 1.17)	2.36 (0.78 ± 0.82)	3.86 (0.88 ± 1.18)
	A-P ant	5.09 (1.95 ± 1.65)	5.53 (1.24 ± 2.01)	4.66 (1.22 ± 1.60)	1.81 (0.67 ± 1.18)	2.36 (0.82 ± 0.96)	2.34 (0.92 ± 1.03)	2.82 (0.98 ± 1.21)	3.10 (1.07 ± 1.36)	3.19 (1.15 ± 1.56)	4.26 (1.06 ± 1.56)
	A-P post		7.13 (3.27 ± 2.40)	6.15 (2.03 ± 1.96)	3.01 (1.24 ± 1.86)	1.94 (0.74 ± 0.62)	1.20 (0.53 ± 0.54)	1.51 (0.57 ± 0.68)	2.16 (0.64 ± 0.87)	2.27 (0.68 ± 0.69)	1.27 (0.50 ± 0.37)
	C-C ant	4.61 (1.86 ± 1.59)	1.69 (0.97 ± 0.81)	3.60 (1.30 ± 1.38)	2.54 (0.83 ± 0.82)	2.45 (0.83 ± 0.75)	2.20 (0.88 ± 0.72)	2.14 (0.84 ± 0.65)	2.09 (0.89 ± 0.63)	2.30 (0.96 ±0.83)	2.61 (0.91 ± 0.78)
	C-C post		3.90 (2.09 ± 1.24)	4.39 (1.57 ± 1.50)	2.41 (0.86 ± 0.70)	1.43 (0.58 ± 0.44)	1.18 (0.50 ± 0.36)	1.17 (0.46 ± 0.38)	1.58 (0.58 ± 0.52)	1.72 (0.67 ± 0.61)	1.59 (0.67 ± 0.54)

The relation between prostate and COM movement was calculated. In Fig. 6a–b, the different sections (anterior and posterior) are represented, and their relation to prostate movement is visualized in R-L, A-P, and C-C directions. The COM deviations from baseline in relation to prostate, which covered 95% of the COM movements, in the R-L direction were 1–4 mm; A-P, 4–7 mm; and C-C, 3–7 mm depending on the section.

### Validation of the deformable registration algorithm

Specific lymph nodes were manually identified in all MRI scans and used as validation structures for the DIR. The difference in lymph node displacement from baseline calculated using DIR and the known deformation from the manually identified lymph nodes was 0.07 ± 0.11 mm (mean ± 1 SD).

## Discussion

It is well known that the prostate is a mobile organ [1–3] and that mobility was confirmed in the present study. Our results also show that the lymph node regions are mobile and can deform. Depending on location in the pelvis, the deformation compared to baseline examination can be over 14 mm in extreme cases.

### Movements

A few studies have evaluated the CTV propagation between treatment planning CT and repeated kV or MV imaging with the patient in treatment position [15–18]. In those studies, lymph nodes had been assumed to be in a stable position in relation to the pelvic bones, i.e., stationary after bone registration. In our present study, deformations were seen in the anterior and the posterior sub-volumes and in different sections. Deformations tended to be larger in the anterior sub-volume than in the posterior sub-volume, and deformations were greater in the sections that were farther away from the prostate (Fig. 5). The largest deformations were observed in the AP direction, and they were fairly equal in the anterior and posterior sub-volumes. Sections close to the prostate usually did not follow the prostate movement pattern. Furthermore, a small prostate deviation from baseline did not imply a small movement in the rest of the CTV.

In a recent study, Lyons et al. estimated CTV-PTV margins by comparing planning CTs to CBCTs [28]. The authors used CBCTs acquired from the first three treatment fractions and thereafter once a week during the rest of the treatment. Volumes were contoured on each CBCT and transferred to the CT with either bone or combined bone and soft tissue registration. CTV volumes for the two registration procedures were compared, and the CTV-PTV margins were calculated using the standard procedure [29]. Margins were found to be 8 mm for soft tissue and 6 mm and for bone registration for lymph nodes. For the prostate, the corresponding results were 5 mm for soft tissue and 8 mm for bone registration. Five CT scans and different margin suggestions were used with the aim to investigate dose coverage for the CTV. The suggested margins were 5 mm around the prostate and 13 mm around the lymph nodes when the prostate image guidance was performed. In conclusion, dose coverage was closely related to margins applied due to the uncertainties in lymph node deformation or daily shifts. Another study, which used CBCT to study daily shifts of the iliac vessels [21] in relation to the prostate, compared three different levels of CTV. Daily shifts of vessels, as a substitute for lymph nodes, showed additional margins due to motion up to 9 mm in the A-P direction and 7 mm in the lateral (R-L) direction, and these margins compensated for daily lymph node displacements relative to the prostate in 95% of the cases.

In this study, we have shown that lymph nodes are mobile with movements over 14 mm from baseline in the A-P direction in some extreme cases. However, in 95% of the cases, the movements are 7 mm or less, depending on the location in the CTV. If we would translate this movement to a margin using [29], systematic and random errors from lymph node motion vary around 1 mm. This gives an indication of the errors in measuring organ motion and a hint about the mobility itself. In most cases, the absolute mean deviations were much lower than the 95% percentile, and this shows the patient-specific movement pattern. The movement is unique for each patient; it is often minor, but in some cases, it can be significant. The larger movements cannot be explained by visual inspection of the images, and they cannot be correlated with large prostate movements (Figs. 4 and 5). The quality assurance procedure using lymph nodes gives validity to the DIR calculation, and the bladder filling protocol results in fairly equal bladder filling at all imaging occasions.

Previous studies mentioned above have been based on CT or CBCT, have used thicker slices (1.5–3 mm), and have produced comparable in-plane resolution, but poorer lymph node visibility due to choice of modality. These studies have used CTV adaptation, or re-drawing, to fit the CTV to the variation in the anatomy between imaging occasions. Two studies used iliac vessels as a surrogate for lymph nodes [21, 30] whereas in this present study, the actual movements of the COM and identified lymph nodes were analyzed. To our knowledge, our present study is the first study to track individual lymph nodes during a radiotherapy course to evaluate movements inside the CTV.

### Prostate vs. bone as reposition landmark in IGRT

We have shown that the prostate and lymph nodes are independently mobile organs (Fig. 5). Today, in clinical routine, there are two common approaches when combining prostate and lymph nodes in the same radiotherapy session, daily reposition according to bone anatomy or to prostate/fiducials. Both approaches treat lymph node as fix to pelvic bone and the margins are adjusted corresponding to repositioning technique. Several studies have suggested margins from these fix node assumptions [16–19]. A recent study using repetitive CT scans and vessels as surrogate for lymph nodes [30] suggests that IGRT based on bony anatomy requires larger prostate and seminal vesicles margins, and guidance on prostate requires larger lymph node margins.

In our study, the movement of COM from baseline can be interpreted as repositions in relation to bone anatomy. In Table 1 and in Fig. 5, we have summarized our findings for the actual movement for prostate and the COM in the different sections in CTV and in different directions. We can notice that the sections containing seminal vesicles (no. 2 and no. 3) are as mobile or even more mobile then prostate in A-P direction. The COM mobility, which covered 95% of the COM movements, in the R-L direction were 1–4 mm; A-P, 1–7 mm; and C-C, 1–5 mm depending on the section.

The COM movement in relation to prostate in our study can be interpreted as IGRT aligning to prostate, Fig.6. The COM movements in relation to prostate, which covered 95% of the cases, in the R-L direction were 1–4 mm, A-P, 4–7 mm; and C-C, 3–7 mm depending on the section. This suggests that only healthy tissue close to prostate gains a prostate landmark approach if only the movements were included in the CTV-PTV margin. Larger margins are needed for all other sections when prostate is used as landmark than if the actual displacement from baseline were used as movement margin. Since the prostate is the main target and the organ at risk (OAR) closest to the prostate receives the highest dose, it is our suggestion that IGRT aligning to prostate is the favored strategy, and larger margins are needed to the lymph nodes to compensate for movement with this approach. We will also stress that the lymph node movement varies across the CTV, and the margins due to lymph node movement vary between 1 and 7 mm depending on direction and location in CTV.

Limitations in this study are the small number of patients (*n* = 10) and the generalized CTV sections. The CTV was divided in up to 40 different sections but due to patient shape and anatomy, the CTV sections may be anatomically slightly different between patients.

## Conclusion

Measurements using repetitive MRI imaging showed that areas inside the CTV (lymph nodes) were as mobile as prostate in some extreme cases and were not fixed to the bones. Both lymph nodes and prostate were observed to be more mobile in the A-P direction than in the C-C or R-L directions. This needs to be considered during treatment planning.

## Electronic supplementary material

ESM 1 (DOCX 13 kb)
