# Supplementary material for: Inter-fraction movements of the prostate and pelvic lymph nodes during IGRT
Source: J Radiat Oncol. 2018 Nov 28;7(4):357–66. doi: 10.1007/s13566-018-0366-3 (PMC6290663; doi:10.1007/s13566-018-0366-3)
Supplement: Supplementary file 1 — (DOCX 13 kb) [file 13566_2018_366_MOESM1_ESM.docx]

## Rigid parameter file:

(FixedInternalImagePixelType "float")

(MovingInternalImagePixelType "float")

(UseDirectionCosines "true")

// **************** Main Components **************************

(Registration "MultiResolutionRegistration")

(Interpolator "BSplineInterpolator")

(ResampleInterpolator "FinalBSplineInterpolator")

(Resampler "DefaultResampler")

(FixedImagePyramid "FixedSmoothingImagePyramid")

(MovingImagePyramid "MovingSmoothingImagePyramid")

(Optimizer "AdaptiveStochasticGradientDescent")

(Transform "EulerTransform")

(Metric "AdvancedMattesMutualInformation")

// ***************** Transformation **************************

(AutomaticScalesEstimation "true")

(AutomaticTransformInitialization "true")

(HowToCombineTransforms "Compose")

// ******************* Similarity measure *********************

(NumberOfHistogramBins 32)

(ErodeMask "false")

// ******************** Multiresolution **********************

(NumberOfResolutions 4)

// ******************* Optimizer ****************************

(MaximumNumberOfIterations 1000)

// **************** Image sampling **********************

(NumberOfSpatialSamples 2048)

(NewSamplesEveryIteration "true")

(ImageSampler "RandomCoordinate")

// ************* Interpolation and Resampling ****************

(FixedImageBSplineInterpolationOrder 1)

(BSplineInterpolationOrder 1)

(FinalBSplineInterpolationOrder 3)

(DefaultPixelValue 0)

(WriteResultImage "true")

(ResultImagePixelType "float")

(ResultImageFormat "mhd")

## Non-Rigid parameter file:

(FixedInternalImagePixelType "float")

(MovingInternalImagePixelType "float")

(FixedImageDimension 3)

(MovingImageDimension 3)

(UseDirectionCosines "true")

// **************** Main Components **************************

(Registration "MultiMetricMultiResolutionRegistration")

(ResampleInterpolator "FinalBSplineInterpolator")

(Resampler "DefaultResampler")

(FixedImagePyramid "FixedGenericImagePyramid" )

(MovingImagePyramid "MovingSmoothingImagePyramid")

(Interpolator "BSplineInterpolator")

(Metric "NormalizedMutualInformation" "TransformBendingEnergyPenalty")

(Metric0Weight 1)

(Metric1Weight 50)

(Optimizer "AdaptiveStochasticGradientDescent")

(Transform "BSplineTransform")

// ******************** Multiresolution **********************

(NumberOfResolutions 3)

(ImagePyramidSchedule 4.0 4.0 4.0 2.0 2.0 2.0 1.0 1.0 1.0)

// ***************** Transformation **************************

(FinalGridSpacingInPhysicalUnits 5 5 5)

(GridSpacingSchedule 4 2 1)

(AutomaticScalesEstimation "true")

(AutomaticTransformInitialization "false")

(HowToCombineTransforms "Compose")

// ******************* Optimizer ****************************

(MaximumNumberOfIterations 300 300 600)

// ******************* Similarity measure *********************

(NumberOfHistogramBins 32 48 48)

(ErodeMask "false")

// **************** Image sampling **********************

(NumberOfSpatialSamples 3000)

(NewSamplesEveryIteration "true")

(ImageSampler "RandomCoordinate")

(CheckNumberOfSamples "true")

(RequiredRatioOfValidSamples 0.01)

(UseRandomSampleRegion "true")

(SampleRegionSize 60 60 60)

(MaximumNumberOfSamplingAttempts 100)

// ************* Interpolation and Resampling ****************

(BSplineInterpolationOrder 2)

(FinalBSplineInterpolationOrder 3)

(DefaultPixelValue -1)

(WriteResultImage "false")

(ResultImagePixelType "short")

(ResultImageFormat "mhd")
